# Ang II Promotes Cardiac Autophagy and Hypertrophy via Orai1/STIM1

**DOI:** 10.3389/fphar.2021.622774

**Published:** 2021-05-17

**Authors:** Chang-Bo Zheng, Wen-Cong Gao, Mingxu Xie, Zhichao Li, Xin Ma, Wencong Song, Dan Luo, Yongxiang Huang, Jichen Yang, Peng Zhang, Yu Huang, Weimin Yang, Xiaoqiang Yao

**Affiliations:** ^1^ School of Pharmaceutical Science and Yunnan Key Laboratory of Pharmacology for Natural Products, Kunming Medical University, Kunming, China; ^2^ School of Biomedical Sciences, The Chinese University of Hong Kong, Shatin, China; ^3^ Longgang E.N.T. Hospital and Shenzhen Key Laboratory of E.N.T., Shenzhen, China

**Keywords:** angiotensin II, autophagy, Cardiac hypertrophy, orai1, STIM1, SOCE

## Abstract

The pathophysiology of cardiac hypertrophy is complex and multifactorial. Both the store-operated Ca^2+^ entry (SOCE) and excessive autophagy are the major causative factors for pathological cardiac hypertrophy. However, it is unclear whether these two causative factors are interdependent. In this study, we examined the functional role of SOCE and Orai1 in angiotensin II (Ang II)-induced autophagy and hypertrophy using *in vitro* neonatal rat cardiomyocytes (NRCMs) and *in vivo* mouse model, respectively. We show that YM-58483 or SKF-96365 mediated pharmacological inhibition of SOCE, or silencing of Orai1 with Orail-siRNA inhibited Ang II-induced cardiomyocyte autophagy both *in vitro* and *in vivo*. Also, the knockdown of Orai1 attenuated Ang II-induced pathological cardiac hypertrophy. Together, these data suggest that Ang II promotes excessive cardiomyocyte autophagy through SOCE/Orai1 which can be the prime contributing factors in cardiac hypertrophy.

## Introduction

The pathophysiology of cardiac hypertrophy is a complex process. It involves dysregulation of multiple cellular factors and/or signaling pathways, including G protein-coupled receptors, autophagy, cytosolic Ca^2+^ signaling, and many others that may contribute to the progression of cardiac hypertrophy (Gupta et al., 2007; Collins et al., 2013).

Autophagy, a highly conserved process, involves the bulk degradation of unnecessary and malfunctioned proteins and organelles (Mizushima et al., 2010). Especially, it plays an essential role in cardiomyocytes which are long-lived differentiated cells. Autophagy helps to maintain cellular homeostasis and healthy cardiomyocyte. However, excessive autophagy can be detrimental, leading to programmed cell death, known as type II cell death (Martinet et al., 2007; Orogo and Gustafsson, 2015). Notably, autophagy has emerged as a key process in the pathogenesis of cardiomyopathies and heart failure (Martinet et al., 2007). For example, excessive myocardial autophagy has been shown to contribute to angiotensin II (Ang II)-induced pathological myocardial hypertrophy in animal models (Porrello et al., 2009; Pan et al., 2013; Kishore et al., 2015; Lin et al., 2016).

Cytosolic Ca^2+^ signaling has been linked to the regulation of autophagy (Decuypere et al., 2011; Parys et al., 2012; Tanwar and Motiani, 2018). However, so far, the majority of autophagy-related studies only focused on the role of intracellular Ca^2+^ release while overlooking the extracellular Ca^2+^ entry. For example, IP3 receptor- and ryanodine receptor-mediated Ca^2+^ release from endo/sarcoplasmic reticulum, and Ca^2+^ release from lysosomes have been shown to regulate autophagy (Zou et al., 2011). In contrast, there are limited reports about the role of extracellular Ca^2+^ entry pathways in autophagy (Sukumaran et al., 2015). Especially, there is no such study in cardiomyocytes.

Store-operated Ca^2+^ entry (SOCE) is a major Ca^2+^ entry pathway in several cell types (Lewis, 2011). In SOCE, depletion of intracellular Ca^2+^ reservoirs stimulates Ca^2+^ entry from the extracellular milieu (Lewis, 2011). The key molecular determinants of SOCE include STIM1 and Orai1. In the process, STIM1 serves as a Ca^2+^ sensor in Sarco/endoplasmic reticulum whereas Orai1 functions as the pore-forming subunit for Ca^2+^-permeation. In cardiomyocytes, L-type Ca^2+^ channels are the principal Ca^2+^ influx channels. Importantly, SOCE is also an important source of Ca^2+^ entry in cardiomyocytes and partly contributes to the Ca^2+^ response against hypertrophic agents Ang II and phenylephrine (Hunton et al., 2002; Zheng et al., 2017). Moreover, excessive Ca^2+^ influx through SOCE could be a causative factor for pathological cardiac hypertrophy (Hunton et al., 2002; Zheng et al., 2017). The pro-hypertrophic role of SOCE has been demonstrated in the pressure overload-induced hypertrophic models (Hulot et al., 2011; Luo et al., 2012) and neurohormonal agent-induced hypertrophic models (Hunton et al., 2002; Ohba et al., 2009; Voelkers et al., 2010; Wang et al., 2015).

Although both SOCE and excessive autophagy are the major pathological causative factors in cardiac hypertrophy, the relationship between them is unclear. In the present study, we explored the functional role of STIM1 and Orai1 in Ang II-induced autophagy in neonatal rat cardiomyocytes (NRCMs). We found that silencing of Orai1 and STIM1 inhibited the pathological autophagy of cardiomyocytes and the mechanism could be related to the Ca^2+^/calmodulin-dependent protein kinase (Ca^2+^-CaMK) signaling pathway.

## Materials and Methods

### Animals

All animal experiments were conducted under the authority of a license issued by the Government of the Yunnan province and approval from the Animal Experimentation Ethics Committee, Kunming Medical University (approval license number: SCXK [滇]K2015–002). Male Sprague-Dawley (S/D) rats (1–2 days old) and male C57BL/6 mice (8 weeks old) were obtained from the Laboratory Animal Services Center of Kunming Medical University.

### Cardiac Hypertrophic Mouse Model

The animal experiments were conducted following the Guide for the Care and Use of Laboratory Animals published by the US National Institute of Health. Eight-week-old male C57 mice were sham-operated or infused with Ang II (1.5 mg/kg/day for 2 weeks) to establish the hypertrophic model using osmotic mini-pumps (Alza Corp, Alzet model 1002, Cupertino, CA, United States). The control mice were infused with physiological saline. 3 days before Ang II treatment, adeno-associated viral 9 vectors carrying Orai1-shRNA (AAV-Orai1-shRNA) and AAV-GFP (Hanhen Crop) were injected into the tail vein of C57 mice (2 × 1012 vg/mice, n = 5). Orai1 shRNA sequence is shown in Supplementary Figure S1A. The silencing efficiency was validated by quantitative real-time PCR (qRT-PCR) and Western blotting (Supplementary Figure S1B–D)

### Cardiac Fibrosis

Cardiac fibrosis assessed as collagen accumulation was determined by picrosirius red staining as described previously (Zheng et al., 2017). Briefly, the heart sections were stained with 0.1% (wt/vol) Sirius red (Sigma, St Louis, MO, United States) in a saturated aqueous solution of picric acid (Wako, Osaka, Japan) for 1 h. After staining, the slides were rinsed with two changes of acidified water [0.5% (wt/wt) glacial acetic acid in H2O], and then dehydrated in three changes of 100% ethanol. The slides were cleared in xylene, mounted in a resinous medium, and then observed under a light microscope. Sirius red-positive areas were measured using the ImageJ software.

### Hypertrophic Model of Cultured Cardiomyocytes

Cardiomyocytes were isolated from 1 to 2-day-old neonatal Sprague-Dawley rats using the conventional method as detailed previously (Wollert et al., 1996). Briefly, rat hearts were dissected and digested with trypsin. The dissociated cells were suspended in Dulbecco’s modified Eagle’s medium [Nutrient Mixture F-12 (DMEM/F12)] supplemented with GlutaMAX (GIBCO, Grand Island, NY, United States, 10565018), 10% horse serum (GIBCO, 16050122), 5% FBS (GIBCO, 11573397), and 50 mg/mL gentamicin (GIBCO, 15710072), and cultured in a humidified incubator (95% air with 5% CO2) for 1 h to allow selective adhesion of cardiac fibroblasts to culture-ware. Non-adhesive cardiomyocytes in suspension were transferred to another dish and cultured for 24 h in a serum-free MEM supplemented with 10 mg/mL transferrin, 10 mg/mL insulin, 1 mg/mL BSA (MEM-TI- BSA), and 0.1 mmol/L BrdU (Zheng et al., 2017). Lastly, the resulting neonatal rat cardiomyocytes (NRCMs) were then treated with 100 nmol/L Ang II for 24 h to induce hypertrophy. On day 2, up to 85% of cells were found α-actinin positive.

### Drugs

SKF-96365 (S7999, Selleck Corporation, United States) (10 μmol/L, soluble in DMSO), YM-58483 (S8380, Selleck Corporation, United States) (3 μmol/L, soluble in DMSO), BAPTA-AM (S7534, Selleck Corporation, United States) (20 μmol/L, soluble in DMSO) and STO-609 (S8274, Selleck Corporation, United States) (10 nmol/L, soluble in DMSO) were added accordingly 12 h before Ang II (100 nmol/L, soluble in water) (Tocris, Bristol, United Kingdom, 1158) application. The culture media containing respective drugs were renewed every 24 h.

### Western Blotting

Total protein was extracted with lysis buffer having 1% (vol/vol) Nonidet P-40, 150 mmol/L NaCl, 20 mmol/L Tris-HCl (pH 7.4), and Roche protease inhibitor cocktail (Sigma–Aldrich, St. Louis, MO, United States, 04693132001). The sample protein concentrations were determined using the DC Protein Assay (Bio-Rad, Hercules, CA, United States, 500–0002). The protein samples were resolved by 12% SDS-PAGE and then transferred onto PVDF membranes. The membranes were blocked with 5% BSA in TBS for 2 h at room temperature (RT), and then overnight incubated with primary antibodies at 4°C. The used primary antibodies were as follows: anti-GAPDH (1:6000, Abcam, Cambridge, United Kingdom, ab8245), anti-Orai1 (1:1000, Sigma–Aldrich, St. Louis, MO, United States, O8264), anti-STIM1 (1:1000, Abcam, ab108994), anti-ANF (1:1000, Abcam, ab14348), anti-β-Actin (1:1000, Abcam, ab14348), and anti-LC3 (1:1000, SIGMA, ab8295). Immunoreactive bands were visualized using the horseradish peroxidase-conjugated secondary antibodies. The intensity of immunoblotted bands was quantified using the ImageJ software.

### siRNA Transfection

NRCMs (1.2 × 106/well) were seeded into the 6-well dish. 24 h after plating, the cells were incubated with 120 nmol/L Orai1-siRNA or 120 nmol/L STIM1-siRNA or 120 nmol/L ATG5-siRNA and 3 μL of RNAiMax (GIBCO, 13778100) in 1 mL of Opti-MEM medium (GIBCO, 51985034) for 6 h. Then, 1 mL of serum-free DMEM containing GlutaMAX was added, followed by an incubation of 18 h. Next, the cells were subjected to Ang II treatment. For the assessment of autophagy as an increase in LC3-II levels, a saturating concentration (10 nmol/L) of bafilomycin A1 was added to the cells 4 h before harvesting. Small interfering RNAs were provided by Hanheng Corp (Orai1-siRNA, L-081151-02-0005, STIM1-siRNA, L-083718-02-0005, and scrambled-siRNA, D-001810-01-05). The silencing efficiency of siRNAs was validated by Western blotting (Supplementary Figure S1G–H)

### Adenovirus-RFP-GFP-LC3 Infection and Fluorescence Microscopy

NRCMs, seeded into 6-well plates (2 × 105 per well), were infected with adenovirus-RFP-GFP-LC3 (10 MOI, Hanheng Corp) by an overnight treatment. The cells were left untreated (control) or treated with adenovirus-GFP for 24 h. After the treatment, the cells were fixed with 4% paraformaldehyde in phosphate-buffered saline (PBS) and maintained at 4°C for immunofluorescence staining. The images were acquired using a Nikon Eclipse 200 fluorescence microscope with MetaMorph software.

### Cytosolic Ca^2+^ Measurement

The cytosolic Ca^2+^ levels were measured as described previously (Qi et al., 2015). Briefly, NRCMs were loaded with 5 μmol/L Fluo-4/AM (Invitrogen, Waltham, MA, United States) for 30 min in normal Tyrode’s solution having 140 NaCl, 5.4 KCl, 1 MgCl_2_, 2 CaCl_2_, 5.5 glucose, and 5 HEPES (all units in mmol/L) at pH 7.4. To deplete intracellular Ca^2+^ stores, the cells were treated with 4 μmol/L thapsigargin (TG) and/or 10 mM/L caffeine (Caf) in Ca^2+^-free Tyrode’s solution. To initiate SOCE, 2.5 mmol/L Ca^2+^ was added to the bath. The real-time fluorescent images were captured every 2 s and analyzed by MetaFluor imaging software (Molecular Devices, United States).

### Statistical Analysis

All values are expressed as mean ± SEM (n), where n denotes the number of independent experiments. The significant differences were determined using paired or unpaired Student's t-test for comparison between the two groups, or one-way ANOVA followed by the Newman--Keuls test for comparison among multiple groups, or two-way ANOVA followed by Bonferroni post-test for comparison in multiple groups. All analyses were performed using the GraphPad Prism software (GraphPad Software, Inc., San Diego, CA, United States). The differences were considered statistically significant at *p* < 0.05.

## Results

### SOCE Promoted Cardiomyocyte Autophagy

**FIGURE 1:**
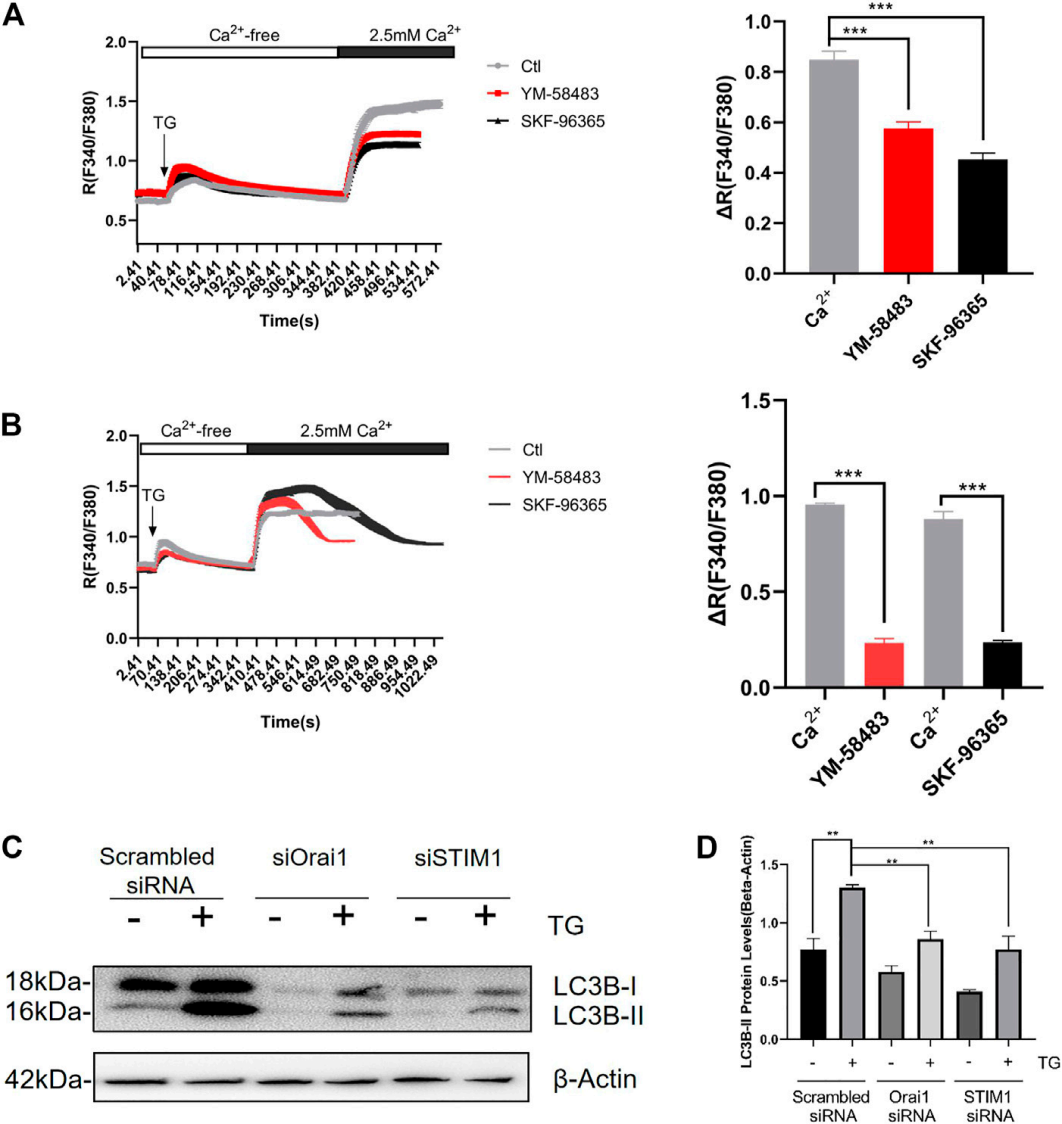
SOCE regulates Ang II-induced autophagy in cardiomyocyte. Acute effect of YM-58483 and SKF-96365 on SOCE in cardiomyocytes. The cells were treated with 4 μmol/L thapsigargin (TG) in a Ca^2+^-free bath to first induce the rise in Ca^2+^, followed by the addition of 2.5 mmol/L Ca^2+^ to induce a second rise in Ca^2+^. The second time rise in Ca^2+^ is due to SOCE **(A)** YM-58483 (3 μmol/L) and SKF-96365 (10 μmol/L) were applied respectively 5 min before TG treatment. The representative traces **(left)** and data summary **(right)** related to Ca^2+^ responses are shown **(B)** YM-58483 (3 μmol/L) and SKF-96365 (10 μmol/L) were correspondingly applied after the addition of 2.5 mmol/L Ca^2+^ to the bath. The representative traces **(left)** and data summary **(right)** of Ca^2+^ responses are shown. Only SOCE is plotted in the summary chart. Knockdown of Orai1/STIM1 attenuated TG-induced autophagy **(C)** NRCMs treated with or without Orai1/STIM1 siRNA were incubated with TG for 24 h. The representative images are showing LC3-II protein levels and summarize data. **p < 0.01, and ***p < 0.001.

We first validated the presence of SOCE. For this, NRCMs were treated with 4 μmol/L thapsigargin (TG) in a Ca^2+^-free bath to deplete intracellular calcium stores. Then, to initiate SOCE, 2.5 mmol/L Ca^2+^ was added to the bath which raised cytosolic Ca^2+^ levels (Figures 1A,B). YM-58483 and SKF-96365 were used as SOCE inhibitors (Yoshino et al., 2007; Chen et al., 2013).

Next, we first added 4 μmol/L TG and then 10 mM/L caffeine (Caf), followed by NRCMs treatment with 3 μmol/L YM-58483 or 10 μmol/L SKF-96365 to inhibit the SOCE (Figure 1A). In other experiments, TG and Caf were added together, then treatment with SOCE inhibitors was performed. Notably, both of these methods showed that SOCE inhibitors (SOCEi) effectively inhibited the Ca^2+^ influx (Supplementary Figure S1A,B).

Furthermore, we investigated the possible involvement of SOCE in cardiomyocyte autophagy. We observed that TG treatment for 24 h induced cardiac autophagy, assessed as an increase in LC3-II protein levels (Figures 1C,D). Fascinatingly, Orai1-specific siRNA-based knockdown of Orai1 dramatically suppressed the TG-induced LC3 II accumulation (Figures 1C,D).

These data suggest that SOCE expressed on NRCMs can induce cardiac autophagy.

### Ang II Promoted Cardiac Autophagy Through SOCE

**FIGURE 2:**
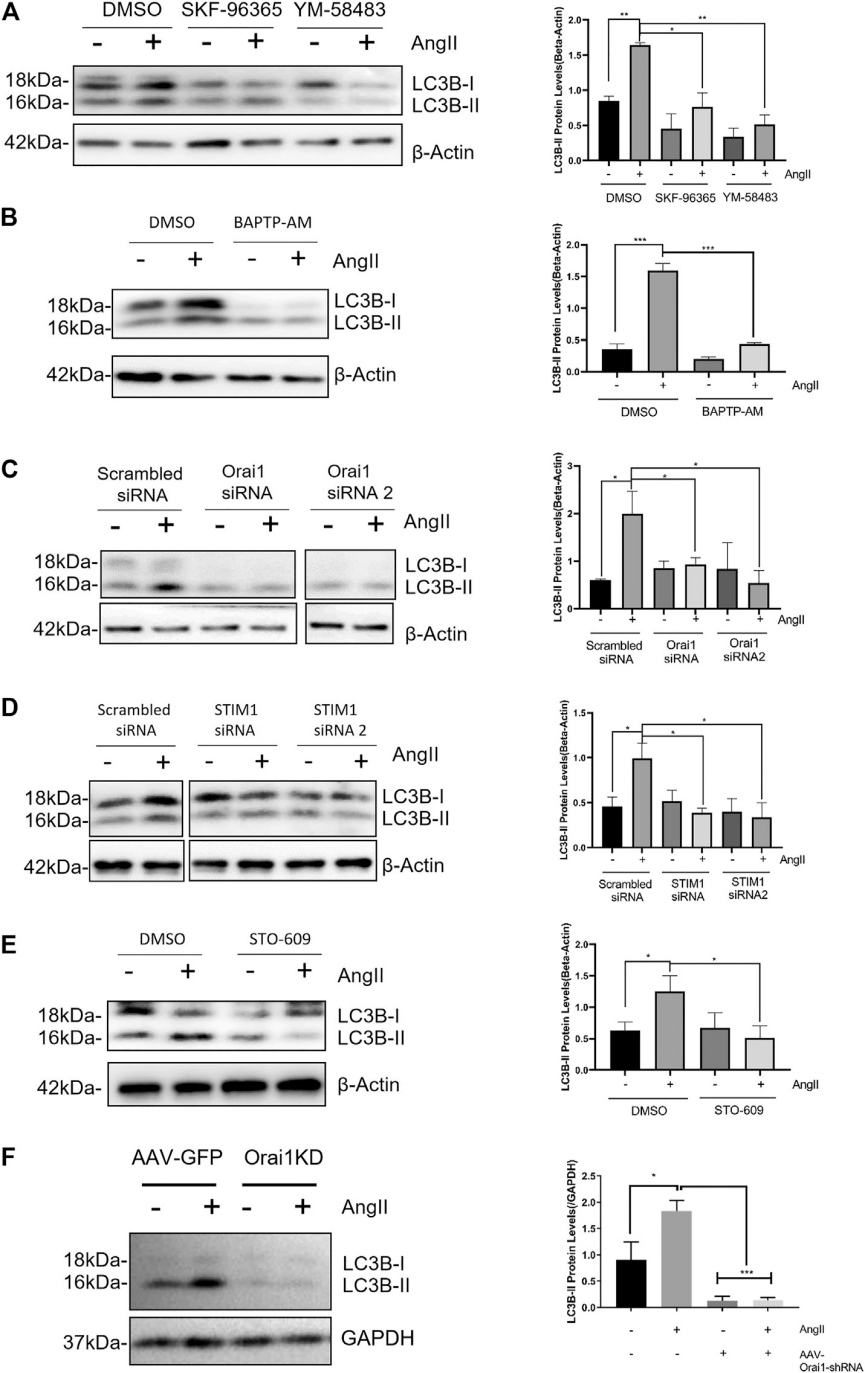
SOCE-calcium-CaMKK is involved in Ang II-induced cardiac autophagy **(A)** Ang II treatment increased LC3-II expression levels in NRCM, which were blocked by SOCE inhibitor YM-58483 and SKF-96365. The representative immunoblots and data summary are shown **(B)** Effect of intracellular calcium chelator BAPTP-AM on LC3-II protein levels in NRCMs. The representative images **(left)** and data summary of immunoblots **(right)** are shown **(C, D)** Representative images and data summary showing that knockdown of Orai1 **(C)** and STIM1 **(D)** in NRCM using Orai1-siRNA and STIM1-siRNA respectively attenuated Ang II-induced cardiac autophagy, as indicated by LC3-II levels **(E)** Effect of CaMKK inhibitor STO-609 on LC3-II protein levels in NRCMs. The representative images **(left)** and data summary of immunoblots **(right)** are shown **(F)** Continuous infusion of Ang II increased LC3-II protein levels in C57 mice, which were blocked by Orai1 knockdown. The representative immunoblots and data summary are shown. *p < 0.05, **p < 0.01, and ***p < 0.001.

Ang II is a commonly used agent for inducing pathological hypertrophy in cardiomyocytes. Ang II-induced excessive autophagy subsequently results in pathological hypertrophy (Pan et al., 2013; Ba et al., 2019; Qi et al., 2020). Therefore, we next examined whether SOCE is involved in Ang II-induced autophagy. As expected, NRCMs treated with 100 nmol/L Ang II for 24 h exhibited increased autophagy. This was evident by an increase in LC3-II levels (Figures 2A–D). Intriguingly, Ang II-induced autophagy was inhibited by SOCE inhibitors YM-58483 (3 μmol/L) or SKF-96365 (10 μmol/L), or chelation of intracellular Ca^2+^ by 20 μmol/L BAPTA-AM (Figures 2A,B). Orail and STIM1 are the known molecular determinants of SOCE. We designed two Orai1-specific siRNAs and two STIM1-specific siRNAs. Both Orai1-siRNA1 and Orai1-siRNA2 effectively reduced the Orai1 expression (Supplementary Figure S1E–H). Importantly, knockdown of Orai1 and STIM1 using corresponding Orai1-siRNAs and STIM1-siRNAs reduced the Ang II-induced autophagy, which was evident by reduced levels of LC3-II (Figures 2C,D). CaMKK is a well-known downstream signal for Orai1-mediated SOCE. We found that inhibition of CaMKK with STO-609 also inhibited the Ang II-induced LC3-II accumulation in NRCMs (Figure 2E).

Next, we established a mouse model of cardiac hypertrophy by continuous infusion of Ang II (1.5 mg/kg/day) in C57 mice for 2 weeks. As expected, Ang II infusion increased LC3-II accumulation in heart tissue suggesting cardiac hypertrophy. Importantly, *in vivo* knockdown of Orai1 using tail injected adeno-associated virus-based Orai1-shRNA (AAV-Orai1-shRNA) markedly reduced Ang II-induced LC3-II accumulation (Figure 2F).

These results, both *in vitro* and *in vivo*, demonstrate that SOCE and Orai1 promote Ang II-induced cardiomyocyte autophagy.

### Orai1 Affected the Induction of Autophagic Flux

**FIGURE 3:**
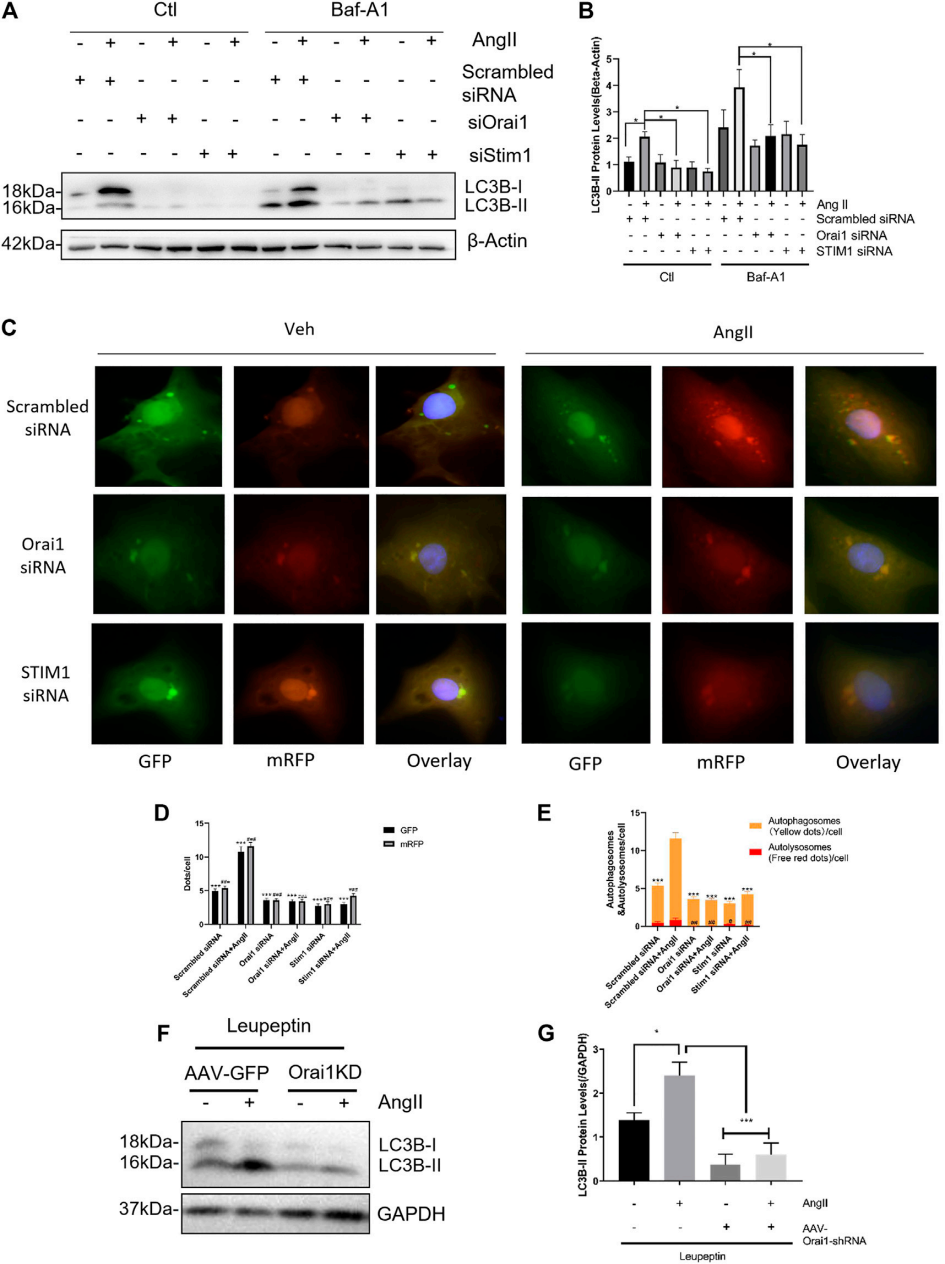
SOCE down-regulates Ang II-induced autophagic flux both *in vivo* and *in vitro.* SOCE siRNAs did not increase the autophagic flux **(A, B)** After bafilomycin A1 (Baf-A1) treatment in NRCM, the representative immunoblots and data summary of LC3B are shown. The effects of Orai1 silencing on LC3-II protein levels in mice hearts with or without Ang II treatment are shown **(C)** NRCMs with or without Orai1 or STIM1 knockdown were transduced with Ad-mCherry-GFP-LC3 for 48 h, followed by culture in normal or glucose-free medium for 3 h. The representative images of GFP and mCherry dots **(C)** together with quantification of autophagosomes and autolysosomes **(D, E)** are shown **(F, G)** C57 mice intraperitoneally (i.p.) injected with leupeptin (40 mg/kg) at the indicated doses every two days were sacrificed after 28 days. The representative immunoblots and data summary of LC3B are shown. *p < 0.05, **p < 0.01, and ***p < 0.001.

Orai1/STIM1 knockdown-based reduction in LC3-II levels could be either due to a decrease in autophagic induction or an increase in autolysosomal degradation. Therefore, to examine that, we performed an autophagic flux assay in cultured NRCMs using bafilomycin A1 (Baf-A1), an inhibitor of lysosome-mediated degradation. We observed that even in the presence of 10 nM bafilomycin A1, Orai1-siRNA1 and STIM1-siRNA could reduce the Ang II-induced LC3-II accumulation (Figures 3A,B).

To further verify the role of Orail in Ang II stimulated autophagic flux, we transfected NRCMs with the adenovirus carrying tandem mRFP-GFP-LC3 construct to effectively and conveniently monitor the autophagic flux (Kimura et al., 2007). In this assay, autophagosomes were labeled with red/green dual fluorescence while autolysosomes were labeled red. We found that Ang II increased the number of both autophagosomes (yellow dots in merged pictures; Figures 3C–E) and autolysosomes (red dots in merged pictures; Figures 3C–E). Notably, Orai1-siRNA reduced the formation of autophagosomes and autolysosomes (Figures 3C–E). Taken together, these data suggest that Orai1/STIM1 knockdown suppressed Ang II-induced autophagic flux.

Similar studies were performed in the mouse model of cardiac hypertrophy as described earlier. Here, we used leupeptin (40 mg/kg, every two days) to inhibit autolysosomal degradation and AAV-Orai1 shRNA for *in vivo* knockdown of Orai1. We observed that in the presence of leupeptin, Ang II infusion markedly increased the LC3-II levels, indicating increased autophagy. However, AAV-Orai1-siRNA1 treatment significantly reduced the Ang II-induced LC3-II accumulation in the mice's heart (Figures 3F,G). These findings suggest that Orai1 could regulate Ang II-induced autophagic flux in the animal model.

### Orai1 as a key Intermediate Signal Contributes to Ang II-Induced Cardiac Hypertrophy

**FIGURE 4:**
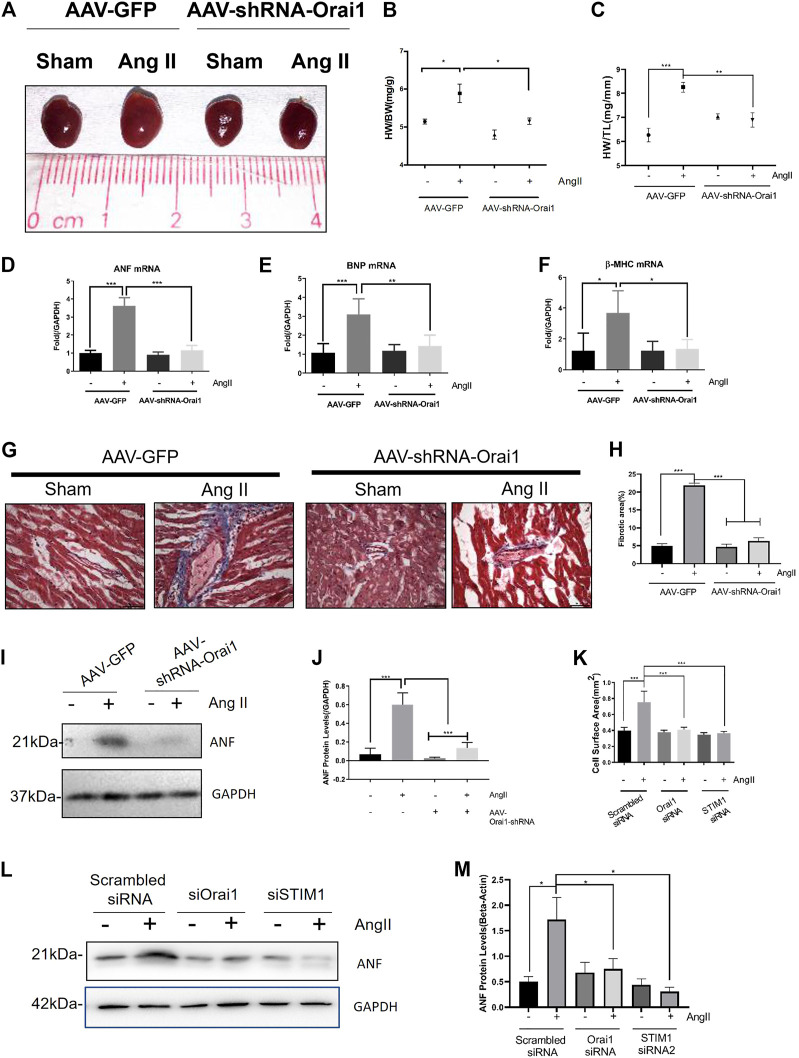
Orai1 knockdown suppresses the Ang II-induced cardiac hypertrophy *in vivo*. Representative images **(A)** and data summary of Heart Weight/Body Weight **(B)** and Heart Weight/Tibia Length **(C)** revealing the effect of AAV-shRNA-Orai1 in the Ang II treatment group (n = 5) and sham group (n = 5) are shown **(D–F)** mRNA levels of hypertrophic markers ANF **(D)**, BNP **(E)**, and β-MHC **(F)** in mice heart. Data are normalized to the vehicle group treated with AAV-GFP **(G, H)** Representative immunoblots **(G)** and data summary **(H)** of Type I collagen fibers in mice hearts revealing the effect of AAV-shRNA-Orai1 in the Ang II treatment group (n = 5) and sham group (n = 5) are shown. Representative images showing AAV-shRNA-Orail mediated reduction in type I collagen levels in the hearts of Ang II treated mice **(I, J)** The immunoblots and data summary of ANF in mice hearts are shown (K–M) Cell surface area of Orai1/STIM1 knockdown **(K)** on Ang-II induced cardiac hypertrophy and the representative immunoblots **(L)** and data summary **(M)** of ANF in NRCM. ns = no significance, *p < 0.05, **p < 0.01, and ***p < 0.001.

We next examined the role of Orai1 by employing the mouse model of Ang II-induced cardiac hypertrophy. To develop cardiac hypertrophy, Ang II (1.5 mg/kg/day) was continuously infused in C57 mice for 2 weeks using the Alzet osmotic pump. As shown in Figures 4A–F, Ang II-treated mice demonstrated an increase in heart size, heart weight/total body weight ratio, heart weight/tibia length, expression of hypertrophic markers (ANF, BNP, and β-MHC), and ANF protein levels (Figures 4I,J). Ang II infusion also led to abnormal accumulation of cardiac collagen fibers (Figures 4G,H). However, tail-vein injection of AAV-Orai1-shRNA (3 days before the Ang II application) alleviated the Ang II-induced heart size enlargement, cardiac fibrosis, and elevation of hypertrophic markers. Moreover, we investigated the impact of Orai1/STIM1 knockdown on Ang II-induced hypertrophy in NRCMs (Figure 4K-M). We found that Orai1/STIM1 siRNA inhibited Ang II-induced cell size enlargement (Figure 4K) and ANP levels (Figures 4L,M). Interestingly, Rapamycin, a potent inducer of autophagy, could reverse the Orai1/STIM1-siRNA induced reduction in ANP levels during Ang II treatment (Supplementary Figure S2A,B). Together, these data suggest that Orai1, as the main mediator of SOCE, promoted Ang II-induced cardiac hypertrophy in the C57 animal model (*in vivo*) and cultured cell (*in vitro*). Moreover, the up-regulated autophagy could reverse the anti-hypertrophic effects of Orai1/STIM1 depletion.

## Discussion

The main findings of this study are as follows: 1) Ang II stimulates autophagy in NRCMs which is evident by an increase in LC3-II accumulation. 2) Ang II-induced autophagy can be substantially reduced by SOCE inhibitors and Orai1/STIM1 silencing. 3) Ang II-induced accumulation of LC3-II was alleviated by chelation of extracellular Ca^2+^ or inhibition of CaMKK. 4) *In vivo* experiments showed that Orai1-siRNA could attenuate Ang II-induced cardiac hypertrophy and fibrosis. Taken together, this study demonstrates the important functional role of Orai1 and SOCE in promoting pathological autophagy and hypertrophy in cardiomyocytes (Supplementary Figure S2G).

Several previous studies explored the possible role of SOCE/STIM1/Orai1 in autophagy using certain cell types including hepatoma cells, prostate cancer cells, and endothelial progenitor cells; however, the conclusions were inconsistent. Some suggested that STIM1/Orai1 stimulates autophagy (Yang et al., 2017; Zhu et al., 2018), while others claimed the opposite (Selvaraj et al., 2016; Tang et al., 2017). It seems that the autophagy-related role of Orai1 and SOCE can be either stimulating or inhibiting depending on the cell type. However, rare studies have examined the role of SOCE/STIM1/Orai1 in cardiomyocyte autophagy (Shaikh et al., 2018). Here, we explored the role of Orai1 in Ang II-induced autophagic flux in NRCMs. We found that Ang II stimulated autophagic flux which was evident by an increase in LC3-II levels in immunoblots and autophagosome formation in RFP-GFP-LC3 tandem reporter assay. Importantly, YM-58483 and SKF-96365 mediated inhibition of SOCE or siRNAs mediated knockdown of Orai1 and STIM1 markedly reduced the Ang II-induced autophagic flux.

Notably, chelation of intracellular Ca^2+^ with BAPTA-AM also abolished the Ang II-induced autophagic flux. This demonstrated that Orai1 and SOCE promoted Ang II-induced autophagy. Furthermore, we explored the downstream signaling pathway of Orai1. A previous report showed that CaMKK could be one of the downstream targets for SOCE/Orai1. In agreement, we also found that STO609, an inhibitor of CaMKK, abolished the Ang II-induced autophagic flux, supporting the notion that CaMKK is situated downstream of Orai1.

Autophagy is a highly complicated process including three key phases, namely induction, substrate targeting, and degradation (Rotter and Rothermel, 2012). An intriguing question is to find a specific autophagic stage (induction or degradation phase) in which Orai1 plays its role. Thus, we used bafilomycin A1 and leupeptin to inhibit vacuolar-type H+ -ATPase, thereby suppressing autophagosomal degradation. However, even in the presence of bafilomycin A1 or leupeptin, Orai1-siRNA could markedly attenuate the Ang II-induced LC3-II accumulation. However, we found that the LC3B-I levels were not consistent in different experiments. One reported that being more sensitive to freezing-thawing and degradation in SDS sample buffer, LC3-I is more labile than LC3-II (Klionsky et al., 2016). The other report suggested that since LC3-II is more sensitive than LC3-I in immunoblotting, a simple comparison of the two, or summation is not appropriate. Rather, comparing the LC3-II levels among different samples is more likely to be an accurate strategy (Mizushima and Yoshimori, 2007). Therefore, we only compared the amounts of LC3-II. Besides, the RFP-GFP-LC3 tandem reporter assay showed that the knockdown of Orai1 reduced the autophagosome formation. Taken together, these findings suggest that Orai1 facilitates autophagy mainly via the induction of autophagic flux.

Our findings have important implications in several heart diseases including pathological cardiac hypertrophy. The pathophysiology of cardiac hypertrophy is complex and multifactorial. Especially, the excessive Ca^2+^ influx through SOCE is known to be one of the causative factors (Hunton et al., 2002; Voelkers et al., 2010; Wang et al., 2015). Accordingly, hypertrophic agents Ang II and phenylephrine (PE) may stimulate excessive Ca^2+^ influx via SOCE, subsequently activating CaMK -calcineurin-NFAT signaling axis triggering pathological cardiac hypertrophy (Hunton et al., 2002; Voelkers et al., 2010). Excessive autophagy is another prime causative factor for cardiac hypertrophy (Martinet et al., 2007; Porrello et al., 2009; Pan et al., 2013; Kishore et al., 2015; Lin et al., 2016). However, it is unknown whether SOCE and autophagy-induced cardiac hypertrophy are the two independent causative factors or these are interlinked. Here, we found that SOCE and Orai1 situate upstream of autophagic signaling cascade to trigger cardiac hypertrophy, involving Orai1-Ca^2+^-autophagy-hypertrophy.

Notably, the majority of published data suggest that excessive Ca^2+^ influx through Orai1/SOCE is a causative factor for pathological cardiac hypertrophy (Hunton et al., 2002; Ohba et al., 2009; Voelkers et al., 2010; Hulot et al., 2011; Luo et al., 2012; Wang et al., 2015; Zheng et al., 2017; Bartoli et al., 2020). Our present data also support this notion. However, conflicting views also exist. Very recently, Segin et al., demonstrated that cardiomyocyte-specific deletion of Orai1 is deleterious in Ang-II-induced cardiac hypertrophy (Segin et al., 2020). This is a contradictory finding from ours. The reason for the data discrepancy is not clear. One possible explanation could be the overcompensation of other channel proteins in Orai1 knockout mice, which was used by Segin et al. (Segin et al., 2020). Another obvious difference is in methodology i.e., we used AAV9-Orai1-shRNA knockdown, which is not a myocardial-specific knockdown, whereas their model was a heart-specific knockout. This makes the comparison more difficult. Further studies are needed to resolve the discrepancy.

In conclusion, our study established an important functional role of SOCE and Orai1 in promoting Ang II-induced autophagy in cardiomyocytes. We suggest that the scheme of Ang II-Orai1-autophagy-hypertrophy may have important pathophysiological relevance in cardiac hypertrophy.

## Data Availability

The raw data supporting the conclusions of this article will be made available by the authors, without undue reservation, to any qualified researcher.

## References

[B1] Ba L. Gao J. Chen Y. Qi H. Dong C. Pan H. (2019). Allicin attenuates pathological cardiac hypertrophy by inhibiting autophagy via activation of PI3K/Akt/mTOR and MAPK/ERK/mTOR signaling pathways. Phytomedicine 58, 152765. 10.1016/j.phymed.2018.11.025 31005720

[B2] Bartoli F. Bailey M. A. Rode B. Mateo P. Antigny F. Bedouet K. (2020). Orai1 channel inhibition preserves left ventricular systolic function and normal Ca 2+ handling after pressure overload. Circulation 141 (3), 199–216. 10.1161/circulationaha.118.038891 31906693 PMC6970549

[B3] Chen T. Zhu J. Zhang C. Huo K. Fei Z. Jiang X. F. (2013). Protective effects of SKF-96365, a non-specific inhibitor of SOCE, against MPP+-Induced cytotoxicity in PC12 cells: potential role of Homer1. PLoS ONE 8 (1), e55601. 10.1371/journal.pone.0055601 23383239 PMC3561331

[B4] Collins H. E. Zhu-Mauldin X. Marchase R. B. Chatham J. C. (2013). STIM1/Orai1-mediated SOCE: Current perspectives and potential roles in cardiac function and pathology. Am J Physiol Heart Circ Physiol. 305 (4), H446-58. 10.1152/ajpheart.00104.2013 23792674 PMC3891250

[B5] Decuypere J.-P. Welkenhuyzen K. Luyten T. Ponsaerts R. Dewaele M. Molgó J. (2011). Ins(1,4,5)P3receptor-mediated Ca2+signaling and autophagy induction are interrelated. Autophagy 7 (12), 1472–1489. 10.4161/auto.7.12.17909 22082873 PMC3327615

[B6] Gupta S. Das B. Sen S. (2007). Cardiac hypertrophy: Mechanisms and therapeutic opportunities. Antioxidants and Redox Signaling. Mary Ann Liebert, Inc. 2 Madison Avenue Larchmont, NY 10538 USA. Antioxid Redox Signal. 9 (6), 623-52. 10.1089/ars.2007.1474 17511580

[B7] Hulot J.-S. Fauconnier J. Ramanujam D. Chaanine A. Aubart F. Sassi Y. (2011). Critical role for stromal interaction molecule 1 in cardiac hypertrophy. Circulation 124 (7), 796–805. 10.1161/circulationaha.111.031229 21810664 PMC3428713

[B8] Hunton D. L. Lucchesi P. A. Pang Y. Cheng X. Dell'Italia L. J. Marchase R. B. (2002). Capacitative calcium entry contributes to nuclear factor of activated T-cells nuclear translocation and hypertrophy in cardiomyocytes. J. Biol. Chem. 277 (16), 14266–14273. 10.1074/jbc.m107167200 11827959

[B9] Kimura S. Noda T. Yoshimori T. (2007). Dissection of the autophagosome maturation process by a novel reporter protein, tandem fluorescent-tagged LC3. Autophagy 3 (5), 452–460. 10.4161/auto.4451 17534139

[B10] Kishore R. Krishnamurthy P. Garikipati V. N. S. Benedict C. Nickoloff E. Khan M. (2015). Interleukin-10 inhibits chronic angiotensin II-induced pathological autophagy. J. Mol. Cell Cardiol. 89 (Pt B), 203–213. 10.1016/j.yjmcc.2015.11.004 26549357 PMC4689660

[B11] Klionsky D. J. Abdelmohsen K. Abe A. Abedin M. J. Abeliovich H. Acevedo Arozena A. (2016). Guidelines for the use and interpretation of assays for monitoring autophagy (3rd edition). Autophagy 12 (1), 1–222. 10.1080/15548627.2016.1139264 26799652 PMC4835977

[B12] Lewis R. S. (2011). Store-operated calcium Channels : new perspectives on mechanism and function. Cold Spring Harbor Perspect. Biol. 3 (12), a003970. 10.1101/cshperspect.a003970 PMC3225942 21791698

[B13] Lin L. Liu X. Xu J. Weng L. Ren J. Ge J. (2016). Mas receptor mediates cardioprotection of angiotensin‐(1‐7) against Angiotensin II‐induced cardiomyocyte autophagy and cardiac remodelling through inhibition of oxidative stress. J. Cel. Mol. Med. 20 (1), 48–57. 10.1111/jcmm.12687 PMC4717848 26515045

[B14] Luo X. Hojayev B. Jiang N. Wang Z. V. Tandan S. Rakalin A. (2012). STIM1-dependent store-operated Ca2+ entry is required for pathological cardiac hypertrophy. J. Mol. Cell Cardiol. 52 (1), 136–147. 10.1016/j.yjmcc.2011.11.003 22108056 PMC3247164

[B15] Martinet W. Knaapen M. W. M. Kockx M. M. De Meyer G. R. Y. (2007). Autophagy in cardiovascular disease. Trends Mol. Med. 13 (11), 482-491. 10.1016/j.molmed.2007.08.004 18029229

[B16] Mizushima N. Yoshimori T. (2007). How to interpret LC3 immunoblotting. Autophagy 3 (6), 542–545. 10.4161/auto.4600 17611390

[B17] Mizushima N. Yoshimori T. Levine B. (2010). Methods in mammalian autophagy research. Cell 140 (3), 313-26. 10.1016/j.cell.2010.01.028 20144757 PMC2852113

[B18] Ohba T. Watanabe H. Murakami M. Sato T. Ono K. Ito H. (2009). Essential role of STIM1 in the development of cardiomyocyte hypertrophy. Biochem. Biophysical Res. Commun. 389 (1), 172–176. 10.1016/j.bbrc.2009.08.117 19715666

[B19] Orogo A. M. Gustafsson Å. B. (2015). Therapeutic targeting of autophagy potential and concerns in treating cardiovascular disease. Circ Res. 116 (3), 489-503. 10.1161/CIRCRESAHA.116.303791 25634972 PMC4313578

[B20] Pan W. Zhong Y. Cheng C. Liu B. Wang L. Li A. (2013). MiR-30-Regulated autophagy mediates angiotensin II-induced myocardial hypertrophy. PLoS ONE 8 (1), e53950. 10.1371/journal.pone.0053950 23326547 PMC3541228

[B21] Parys J. B. Decuypere J. P. Bultynck G. (2012). Role of the inositol 1,4,5-trisphosphate receptor/Ca2+-release channel in autophagy. Cell Commun Signal. 10 (1), 17. 10.1186/1478-811X-10-17 22770472 PMC3413604

[B22] Porrello E. R. D'Amore A. Curl C. L. Allen A. M. Harrap S. B. Thomas W. G. (2009). Angiotensin II type 2 receptor antagonizes angiotensin ii type 1 receptor-mediated cardiomyocyte autophagy. Hypertension 53 (6), 1032–1040. 10.1161/hypertensionaha.108.128488 19433781

[B23] Qi H. Ren J. Ba L. Song C. Zhang Q. Cao Y. (2020). MSTN attenuates cardiac hypertrophy through inhibition of excessive cardiac autophagy by blocking AMPK/mTOR and miR-128/pparγ/NF-κB. Mol. Ther. - Nucleic Acids 19, 507–522. 10.1016/j.omtn.2019.12.003 31923740 PMC6951838

[B38] Qi Y. Qi Z. Li Z. Wong C.‐K. So C. Lo I.‐C. (2015). Role of TRPV1 in the differentiation of mouse embryonic stem cells into Cardiomyocytes. Plos one 10 (7), e0133211. 10.1371/journal.pone.0133211 26208267 PMC4514823

[B24] Rotter D. Rothermel B. A. (2012). Targets, trafficking, and timing of cardiac autophagy. Pharmacol. Res. 66 (6):494-504. 10.1016/j.phrs.2012.10.001 23059539 PMC3502698

[B25] Segin S. Berlin M. Richter C. Flockerzi R. M. V. Worley P. Freichel M. (2020). Cardiomyocyte-specific deletion of Orai1 reveals its protective role in angiotensin-II-induced pathological cardiac remodeling. Cells 9 (5), 1092. 10.3390/cells9051092 PMC7290784 32354146

[B26] Selvaraj S. Sun Y. Sukumaran P. Singh B. B. (2016). Resveratrol activates autophagic cell death in prostate cancer cells via downregulation of STIM1 and the mTOR pathway. Mol. Carcinog. 55 (5), 818–831. 10.1002/mc.22324 25917875 PMC4624064

[B27] Shaikh S. Troncoso R. Mondaca-Ruff D. Parra V. Garcia L. Chiong M. (2018). The STIM1 inhibitor ML9 disrupts basal autophagy in cardiomyocytes by decreasing lysosome content. Toxicol. Vitro 48, 121–127. 10.1016/j.tiv.2018.01.005 29337250

[B28] Sukumaran P. Sun Y. Vyas M. Singh B. B. (2015). TRPC1-mediated Ca2+ entry is essential for the regulation of hypoxia and nutrient depletion-dependent autophagy. Cell Death Dis 6 (3), e1674. 10.1038/cddis.2015.7 25741599 PMC4385947

[B29] Tang B.-D. Xia X. Lv X.-F. Yu B.-X. Yuan J.-N. Mai X.-Y. (2017). Inhibition of Orai1-mediated Ca2+entry enhances chemosensitivity of HepG2 hepatocarcinoma cells to 5-fluorouracil. J. Cel. Mol. Med. 21 (5), 904–915. 10.1111/jcmm.13029 PMC5387165 27878958

[B30] Tanwar J. Motiani R. K. (2018). Role of SOCE architects STIM and orai proteins in cell death. Cell Calcium. 69:19-27. 10.1016/j.ceca.2017.06.002 28629579

[B31] Voelkers M. Salz M. Herzog N. Frank D. Dolatabadi N. Frey N. Gude N. Friedrich O. Koch W. J. Katus H. A. Sussman M. A. Most P. (2010). Most, P.Orai1 and Stim1 regulate normal and hypertrophic growth in cardiomyocytes. J. Mol. Cell Cardiol. 48 (6), 1329–1334. 10.1016/j.yjmcc.2010.01.020 20138887 PMC5511312

[B39] Wollert K. C. Taga T. Saito M. Narazaki M. Kishimoto T. Glembotski C. C. (1996). Cardiotrophin-1 activates a distinct form of cardiac muscle cell hypertrophy. Assembly of sarcomeric units in series VIA gp130/leukemia inhibitory factor receptor-dependent pathways. J. Biol. Chem. 271 (16), 9535–9545. 10.1074/jbc.271.16.9535 8621626

[B32] Wang Y. Li Z. C. Zhang P. Poon E. Kong C. W. Boheler K. R. (2015). Nitric oxide-cGMP-PKG pathway acts on Orai1 to inhibit the hypertrophy of human embryonic stem cell-derived cardiomyocytes. Stem Cells 33 (10), 2973–2984. 10.1002/stem.2118 26269433

[B33] Yang J. Yu J. Li D. Yu S. Ke J. Wang L. (2017). Store-operated calcium entry-activated autophagy protects EPC proliferation via the CAMKK2-MTOR pathway in ox-LDL exposure. Autophagy 13 (1), 82–98. 10.1080/15548627.2016.1245261 27791458 PMC5240837

[B34] Yoshino T. Ishikawa J. Ohga K. Morokata T. Takezawa R. Morio H. (2007). YM-58483, a selective CRAC channel inhibitor, prevents antigen-induced airway eosinophilia and late phase asthmatic responses via Th2 cytokine inhibition in animal models. Eur. J. Pharmacol. 560(2–3), 225–233. 10.1016/j.ejphar.2007.01.012 17307161

[B35] Zheng C. Lo C. Y. Meng Z. Li Z. Zhong M. Zhang P. (2017). Gastrodin inhibits store-operated Ca2+entry and alleviates cardiac hypertrophy. Front. Pharmacol. 8 (APR), 222. 10.3389/fphar.2017.00222 28487655 PMC5404510

[B36] Zhu Z. D. Yu T. Liu H. J. Jin J. He J. (2018). SOCE induced calcium overload regulates autophagy in acute pancreatitis via calcineurin activation article. Cel Death Dis. 9 (2), 1–12. 10.1038/s41419-017-0073-9 PMC5833430 29352220

[B37] Zou Y. Liang Y. Gong H. Zhou N. Ma H. Guan A. (2011). Ryanodine receptor type 2 is required for the development of pressure overload-induced cardiac hypertrophy. Hypertension 58 (6), 1099–1110. 10.1161/hypertensionaha.111.173500 21986507

