# Supplementary figures and images for: Ang II Promotes Cardiac Autophagy and Hypertrophy via Orai1/STIM1
Source: Front Pharmacol. 2021 May 17;12:622774. doi: 10.3389/fphar.2021.622774 (PMC8165566; doi:10.3389/fphar.2021.622774)

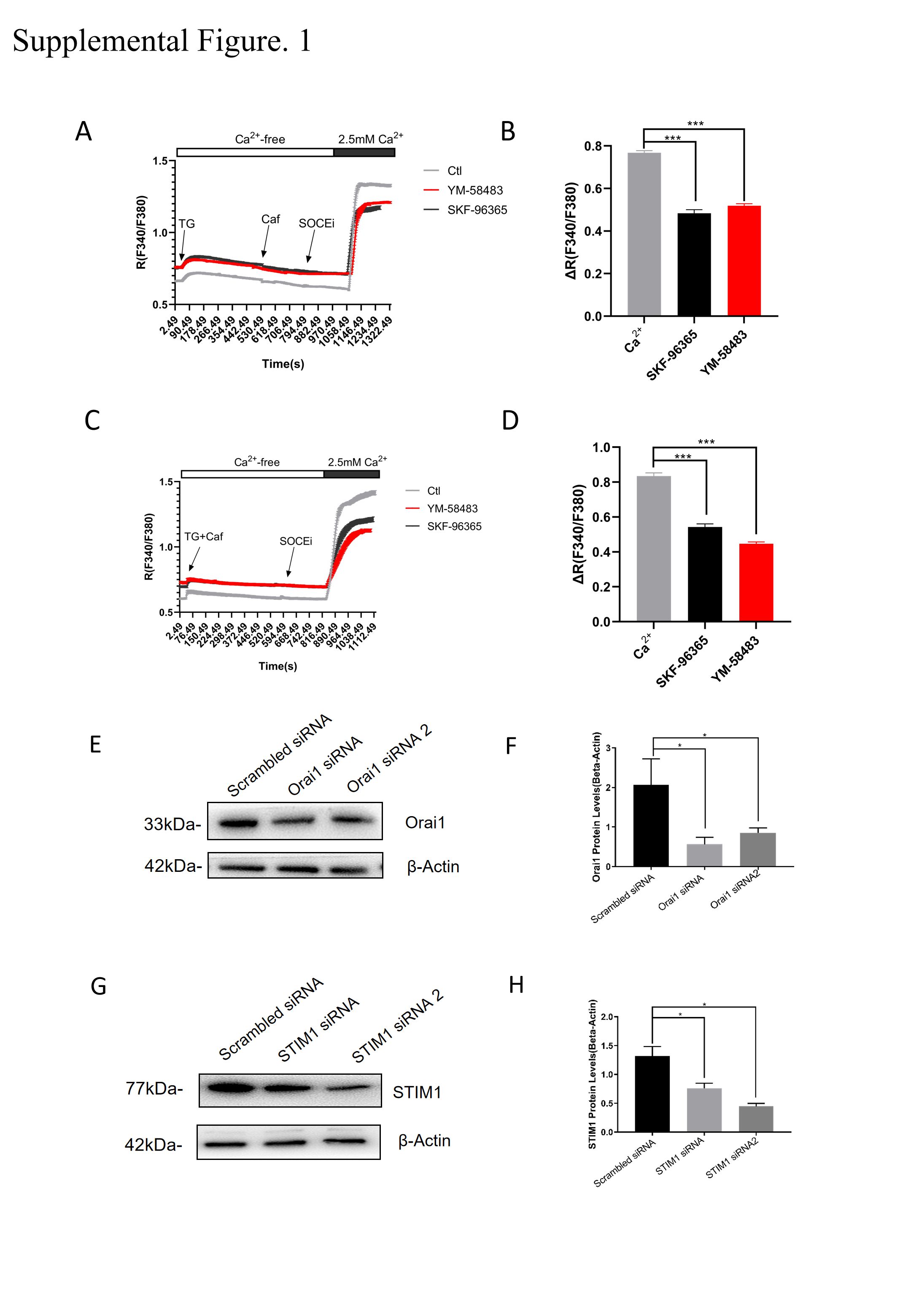

Supplement: Supplementary file 1 [file Image1.jpeg]

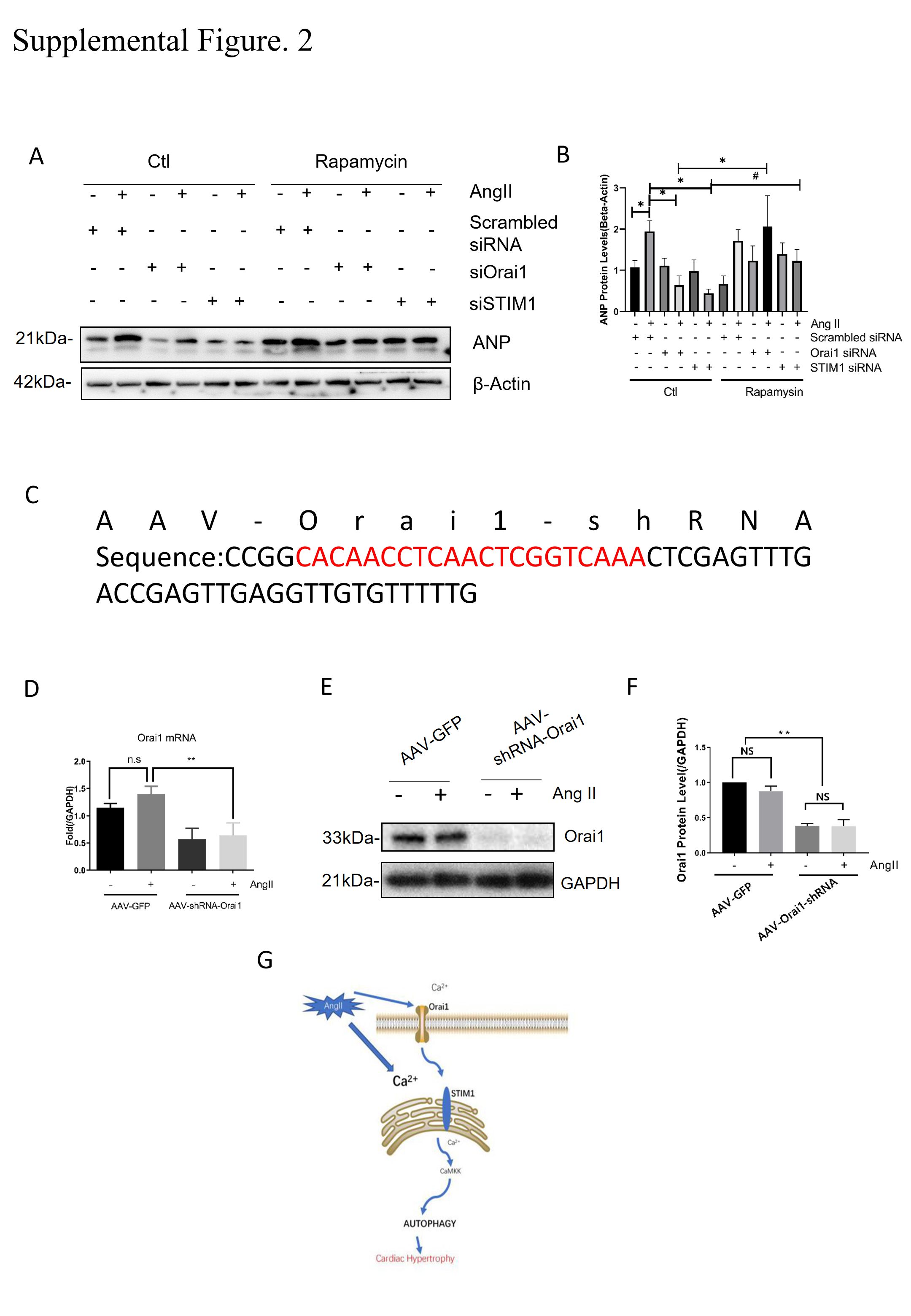

Supplement: Supplementary file 2 [file Image2.jpeg]
